# The role of sulfonate groups and hydrogen bonding in the proton conductivity of two coordination networks

**DOI:** 10.3762/bjnano.13.36

**Published:** 2022-05-04

**Authors:** Ali Javed, Felix Steinke, Stephan Wöhlbrandt, Hana Bunzen, Norbert Stock, Michael Tiemann

**Affiliations:** 1 Department of Chemistry, Paderborn University, 33098 Paderborn, Germany; 2 Institute of Inorganic Chemistry, Christian-Albrecht University, Kiel, Germany; 3 Institute of Physics, University of Augsburg, 86159 Augsburg, Germany

**Keywords:** coordination network, coordination polymer, impedance spectroscopy, metal-organic framework, proton conductivity

## Abstract

The proton conductivity of two coordination networks, [Mg(H_2_O)_2_(H_3_L)]·H_2_O and [Pb_2_(HL)]·H_2_O (H_5_L = (H_2_O_3_PCH_2_)_2_-NCH_2_-C_6_H_4_-SO_3_H), is investigated by AC impedance spectroscopy. Both materials contain the same phosphonato-sulfonate linker molecule, but have clearly different crystal structures, which has a strong effect on proton conductivity. In the Mg-based coordination network, dangling sulfonate groups are part of an extended hydrogen bonding network, facilitating a “proton hopping” with low activation energy; the material shows a moderate proton conductivity. In the Pb-based metal-organic framework, in contrast, no extended hydrogen bonding occurs, as the sulfonate groups coordinate to Pb^2+^, without forming hydrogen bonds; the proton conductivity is much lower in this material.

## Introduction

Recent achievements in the synthesis of advanced functional materials with tailored, structure-related physical properties have stimulated the development of new concepts and devices for energy storage [1–2] and energy conversion [3–4]. Among these, proton-conducting solid materials show significant potential in the development of novel membranes for proton exchange membrane (PEM) fuel cells, PEM electrolyzers, and for humidity sensors [5–7]. The goal is to overcome the restrictions of state-of-the-art proton-conducting membrane materials such as Nafion. Despite its high proton conductivity of ca. 0.1 S·cm^−1^ at 80 °C and 98% relative humidity (r.h.), Nafion exhibits some shortcomings, including high cost, sophisticated manufacturing processes, and stringent operating conditions [8]. Therefore, a variety of new functional network materials are currently being discussed as potential alternatives. These include coordination polymers (CPs), covalent organic frameworks (COFs), polyoxometalates (POMs), hydrogen-bonded organic frameworks (HOFs), and mesoporous organosilica materials (MPOs) [3,9–11]. In particular, proton-conducting coordination polymers (CPs), such as (porous) metal-organic frameworks (MOFs) and (non-porous, yet cross-linked) coordination networks [12], may offer alternatives to Nafion because of their structural controllability and high crystallinity [13].

The quest to develop new proton-conducting network materials is associated with the requirement to understand conduction mechanisms and to identify the necessary characteristics of the crystal structures of the materials [14]. This will offer new options to design structures with appropriate functionalities, such as (i) by introducing or replacing functional groups of the bridging ligands, for example, –OH, –COOH, –PO_3_H_2_ or –SO_3_H, (ii) by filling pores or channels with acidic guest molecules, such as oxonium ions, organic or inorganic acids, or ammonium cations, or (iii) by ligand substitution to increase the mobility of proton carriers [13–16]. Here we report an investigation of proton conductivity in two coordination networks that contain the same phosphonato-sulfonate linker, but different metal nodes, namely Pb^2+^ and Mg^2+^ [17]. The materials exhibit different crystal structures, and both show a certain, though moderate, proton conductivity that is strongly humidity- and temperature-dependent. We discuss how differences in the conduction behavior may be related to the respective structural properties.

## Results and Discussion

**Figure 1:**
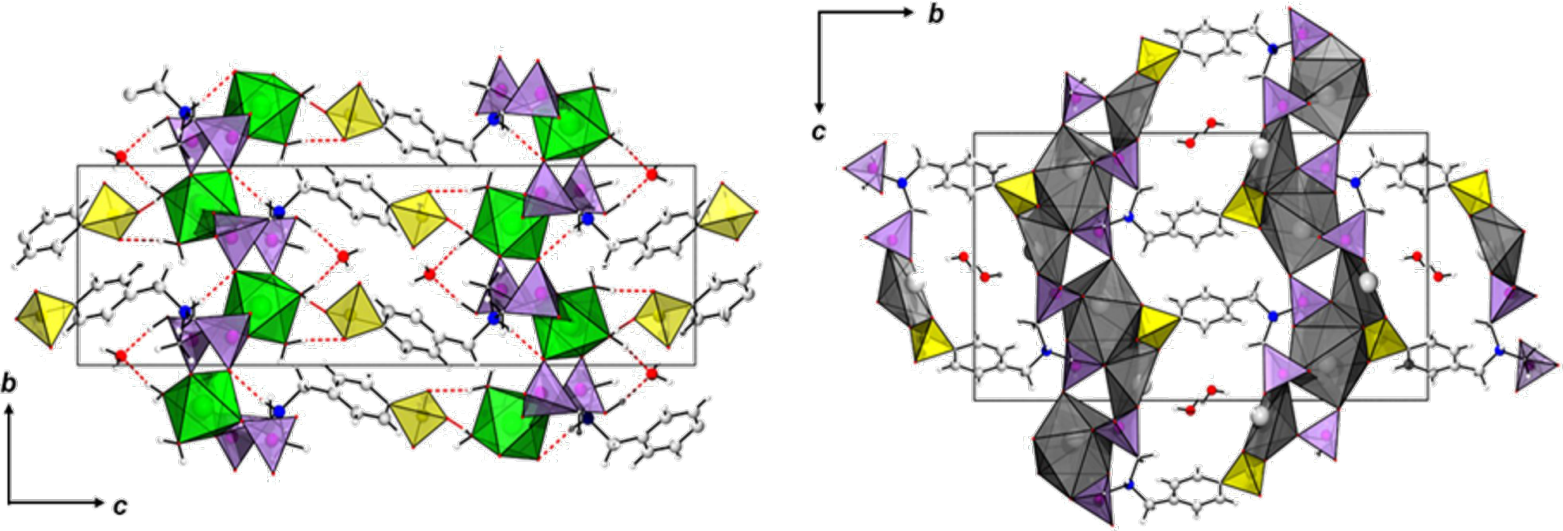
Crystal structures of [Mg(H_2_O)_2_(H_3_L)]·H_2_O) (left) and [Pb_2_(HL)]·H_2_O) (right). General color scheme: Mg: green octahedra, Pb: dark grey polyhedra, P: violet tetrahedra, S: yellow tetrahedra, C: light grey, N: blue, H: white, H bonds: red dotted lines.

The compounds [Mg(H_2_O)_2_(H_3_L)]·H_2_O and [Pb_2_(HL)]·H_2_O were synthesized using the linker molecule H_5_L (see Supporting Information File 1, Figure S1) and magnesium chloride hexahydrate (MgCl_2_⋅6H_2_O) or lead acetate hydrate (Pb(OAc)_3_·H_2_O), respectively, under solvothermal reaction conditions, employing mixtures of water and ethanol as solvent. In the case of [Mg (H_2_O)_2_(H_3_L)]·H_2_O, sodium hydroxide was used as an additive [13]. Crystallographic and experimental details, as well as an extended structure characterization and discussion is provided in [17]. In brief, both compounds crystallize in the same monoclinic space group (*P*2_1_/*c*, Nr. 14) but exhibit fundamentally different crystal structures (Figure 1). [Mg(H_2_O)_2_(H_3_L)]·H_2_O is a 2D coordination network (I^2^O^0^ network following the classification by Cheetham et al. [18]), with a 2D inorganic building unit consisting of corner-sharing CPO_3_ and MgO_6_ polyhedra. Each Mg^2+^ ion is surround by four different phosphonate groups and two aqua ligands, with the phosphonate groups bridging two metal ions, resulting in the formation of layers. This way, each phosphonate group is coordinating with two oxygen atoms, while the third one is protonated. The –C_6_H_4_-SO_3_ group points into the interlayer space. A network of hydrogen bonds between the sulfonate residues and coordinated water molecules as well as crystal water interconnect the layers into a three-dimensional network consisting of interlocked layers. In contrast, in the structure of [Pb_2_(HL)]·H_2_O, each sulfonate group is coordinating to several Pb^2+^ ions. A 2D inorganic building unit is observed and the coordination of the sulfonate groups results in the formation of a 3D coordination network (I^2^O^1^) exhibiting ultramicropores (2 × 4 Å) [17]. Therefore, in contrast to the magnesium-based compound, [Pb_2_(HL)]·H_2_O can be denoted as a metal-organic framework [12]. It is noteworthy that solely H bonds with adsorbed water molecules are found. Crystallographic and experimental details, as well as an extended structure discussion is provided in [17].

**Figure 2:**
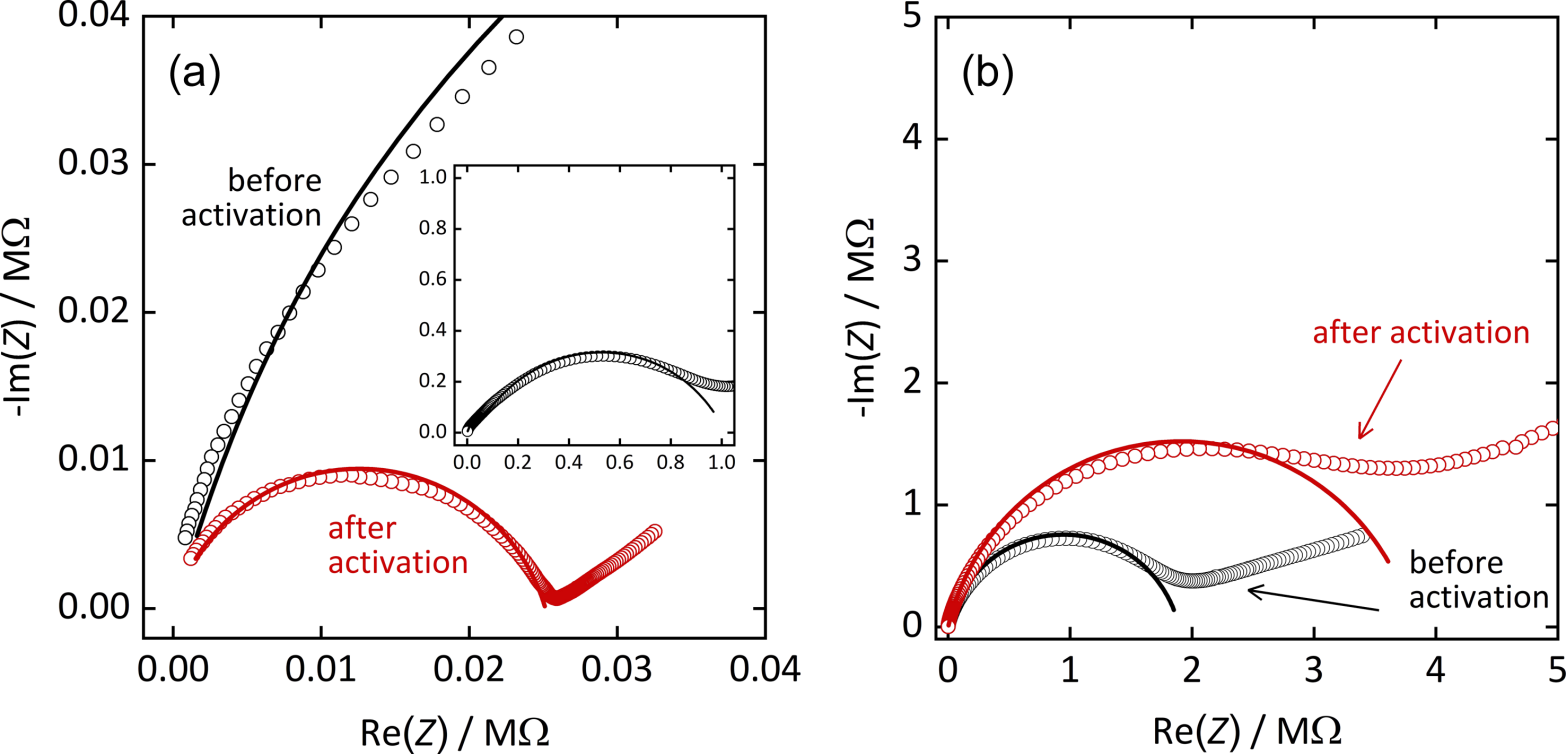
Nyquist plots (imaginary vs real part of impedance *Z*) of (a) [Mg(H_2_O)_2_(H_3_L)]·H_2_O and (b) [Pb_2_(HL)]·H_2_O, before and after thermal activation. Measurements were conducted at 22 °C and 90% r.h.; lines represent the fit results from using the equivalent circuits described in the text.

**Figure 3:**
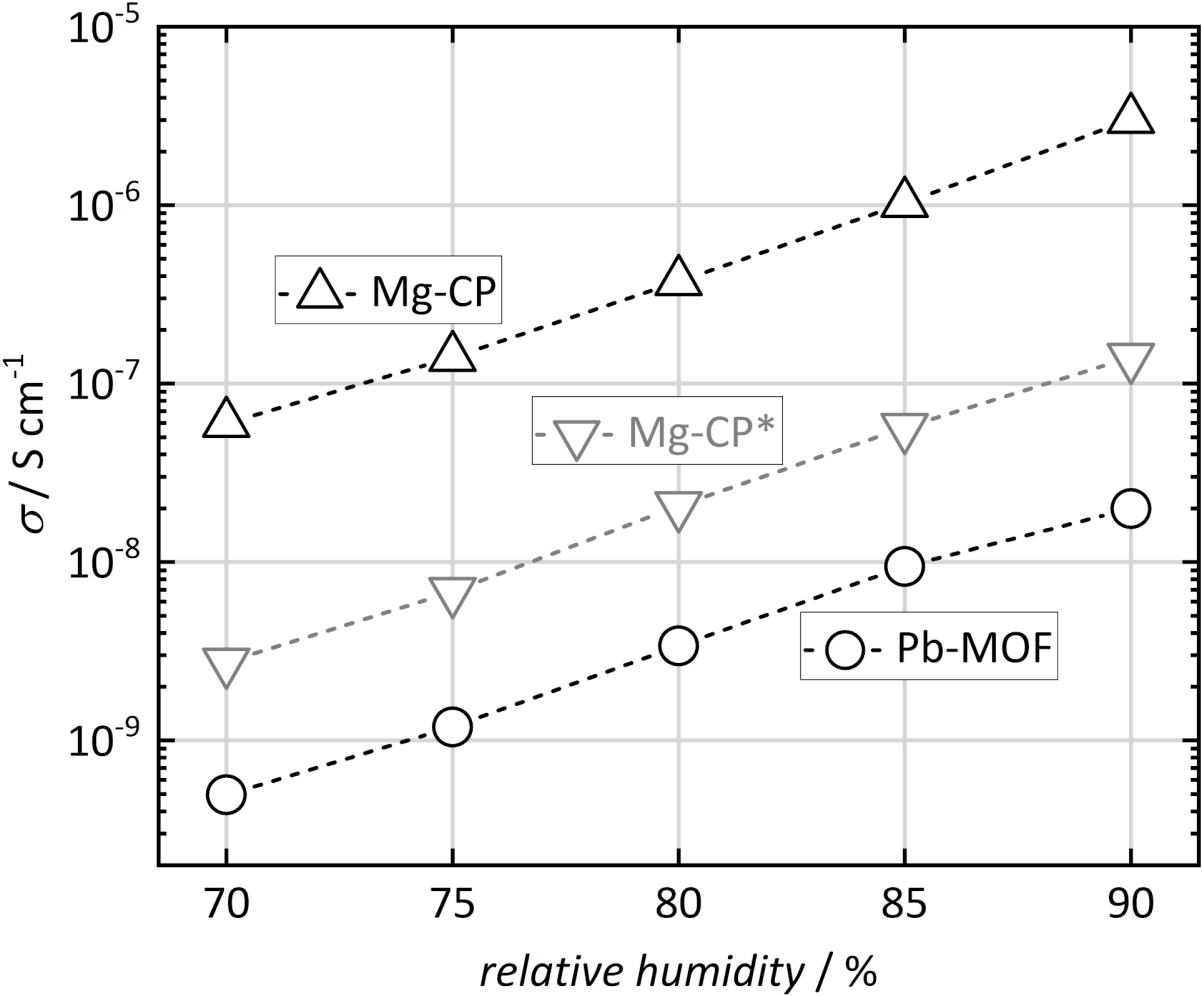
Proton conductivity (dashed lines as a guide to the eye) of [Mg(H_2_O)_2_(H_3_L)]·H_2_O (Mg-CP) and [Pb_2_(HL)]·H_2_O (Pb-MOF) at variable relative humidity (22 °C, before thermal activation). *The lower values for Mg-CP (gray) correspond to the conduction mode that is irreversibly erased by thermal activation.

The proton conductivity of [Mg(H_2_O)_2_(H_3_L)]·H_2_O (in the following: Mg-CP) and [Pb_2_(HL)]·H_2_O (in the following: Pb-MOF) was investigated by AC impedance analysis [19–20]. For this purpose, the materials were pressed into pellets and placed between plate electrodes without further auxiliary measures, such as the usage of conductive paste. A potential of 0.1 V was chosen for the entire study, after making sure that lower or higher potentials do not affect the measurements in any significant way (see Supporting Information File 1, Figure S2). Figure 2 shows the Nyquist plots of the impedance spectra (i.e., imaginary vs real part of the impedance *Z*) for both materials at 22 °C and 90% r.h., before and after thermal activation. The term “thermal activation” here stands for exposure to 80 °C at ambient pressure for 24 h in dry air. This process is supposed to remove surface-adsorbed water, such as residues from the synthesis process or from exposure to humid conditions. Powder X-ray diffraction confirmed that this process does not affect the crystallinity of the two materials, and no phase transformation was observed (see Supporting Information File 1, Figure S3 and Figure S4). The Nyquist plots show depressed semicircles in the high-frequency region (i.e., region of low Re(*Z*)) that allow for the determination of the proton conductance by fitting an appropriate equivalent circuit model. Here, the model consists of a resistor and a parallel constant phase element (Supporting Information File 1, Figure S5a). However, in case of the non-activated Mg-CP sample, a second pair of resistor and parallel constant phase element, serial to the first pair (Supporting Information File 1, Figure S5b), was found to be necessary, as will be discussed below. The proton conductivity σ is then calculated from the resistance *R* by accounting for the thickness *L* and contact area *A* of the sample pellet between the two electrodes (σ = *R*^−1^*·L·A*^−1^) [21]. The results are shown in Table 1. Further conductivity values at 22 °C are plotted in Figure 3 for r.h. values between 70% and 90%. A strong impact of humidity is observed for both materials; obviously, proton conduction is strongly mediated by water, as frequently observed for similar materials [22–23].

**Table 1:** Proton conductivity of [Mg(H_2_O)_2_(H_3_L)]·H_2_O (Mg-CP) and [Pb_2_(HL)]·H_2_O (Pb-MOF), before and after thermal activation.

	before thermal activation		after thermal activation	
	
	σ/S·cm^−1^	*E*_A_/eV	σ/S·cm^−1^	*E*_A_/eV

Pb-MOF	2.0 × 10^−8^	0.17 ± 0.02	9.9 × 10^−9^	0.36 ± 0.04
Mg-CP	3.0 × 10^−6^ (1.40 × 10^−7^)^a^	0.40 ± 0.05 (0.64 ± 0.01)^a^	3.6 × 10^−6^	0.38 ± 0.05

^a^The second values correspond to the conduction mode that is irreversibly erased by thermal activation.

The Pb-MOF sample shows an overall very low proton conductivity. The value increases from 4.9 × 10^−10^ S·cm^−1^ at 70% r.h. to 2.0 × 10^−8^ S·cm^−1^ at 90% r.h. (non-activated sample, 22 °C). Activation only leads to a very slight change in conductivity (9.9 × 10^−9^ S·cm^−1^ at 90% r.h., see Table 1). These findings suggest that proton mobility is mostly governed by the (reversible) uptake of water, either inside or outside the pores of Pb-MOF.

**Figure 4:**
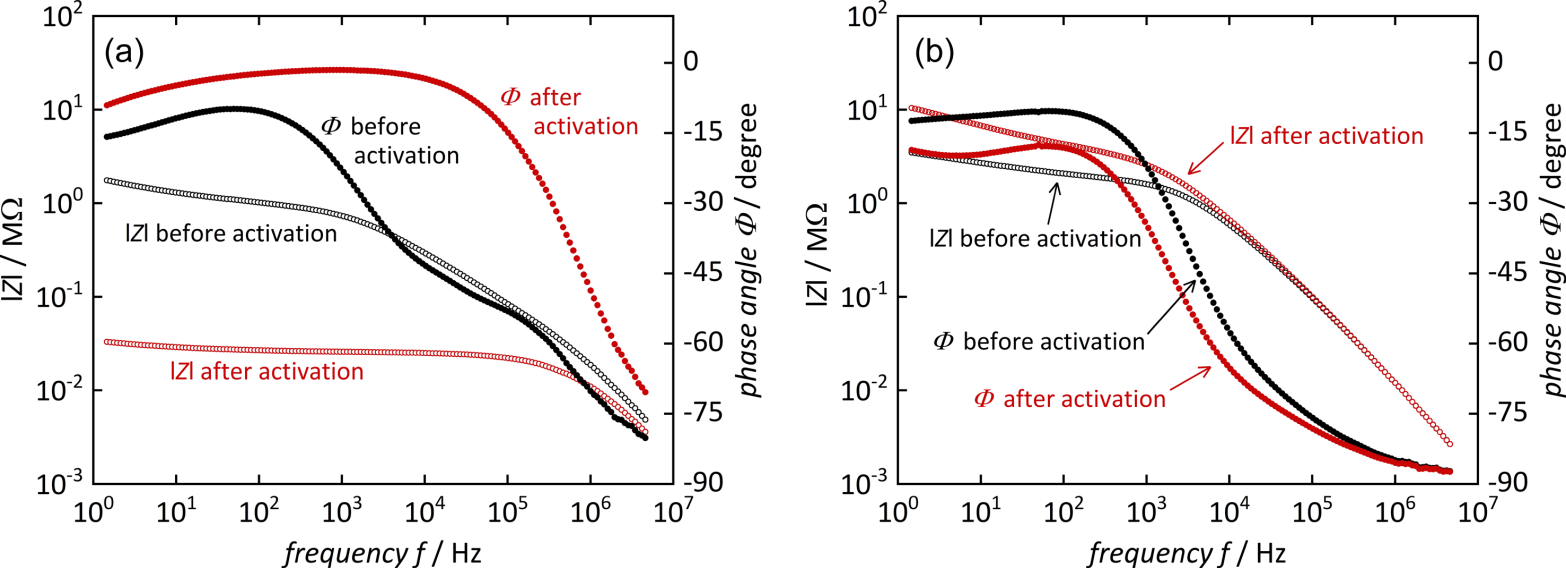
Bode plots (absolute value of impedance *Z* and phase angle Φ vs frequency *f*) of (a) [Mg(H_2_O)_2_(H_3_L)]·H_2_O and (b) [Pb_2_(HL)]·H_2_O, before and after thermal activation. Measurements were conducted at 22 °C and 90% r.h.

The Mg-CP sample shows a more interesting behavior. Its conductivity lies in the range between 6 × 10^−8^ S·cm^−1^ (70% r.h.) and 3 × 10^−6^ S·cm^−1^ (90% r.h.), which are still only low values, but approximately two orders of magnitude higher than those of Pb-MOF. (A brief survey of proton conductivity values in other coordination networks containing sulfonate groups, extracted from literature, is shown in Supporting Information File 1, Table S1) For the non-activated Mg-CP sample, it turns out that a satisfying fit of the Nyquist plot data is obtained by using a modified equivalent circuit model, as mentioned above. A second semicircle, superimposing the first one, needs to be accounted for, though this is hardly distinguishable by the naked eye. This finding suggests that, in this case, proton conduction occurs by two distinct processes. The second one is irreversibly erased by thermal activation; the additional semicircle is no longer observed and, hence, the standard equivalent circuit model can be applied once the sample has been activated. This is further illustrated in Figure 4, which shows the Bode plots (i.e., impedance *Z* and phase angle Φ vs frequency *f*) of the measurements from Figure 2. For the non-activated Mg-CP sample, the phase angle shows two distinct features, namely a maximum at ca. 10^2^ Hz and a shoulder at ca. 10^5^ Hz, corresponding to two distinguishable conduction modes. After thermal activation, only a single broad maximum in the region from 10^3^ to 10^4^ Hz remains. The phase angle for the Pb-MOF sample shows only one maximum (at ca. 10^2^ Hz) both before and after activation. We conclude that the second conductance mode in the non-activated Mg-CP material may be caused by interparticle water adsorbate layers that are successfully and irreversibly removed by activation. These findings are quite helpful, as they allow us to conversely conclude that the remaining mode of proton conductance is probably not caused by such adsorbate layers, but more likely occurs within the crystalline material, as will be elaborated in more detail below. (We did not observe any semicircular behavior in the absence of humidity, neither before nor after thermal activation, from which we conclude that other phenomena, such as relaxation of electric dipoles, do not significantly contribute to the charge mobility.)

**Figure 5:**
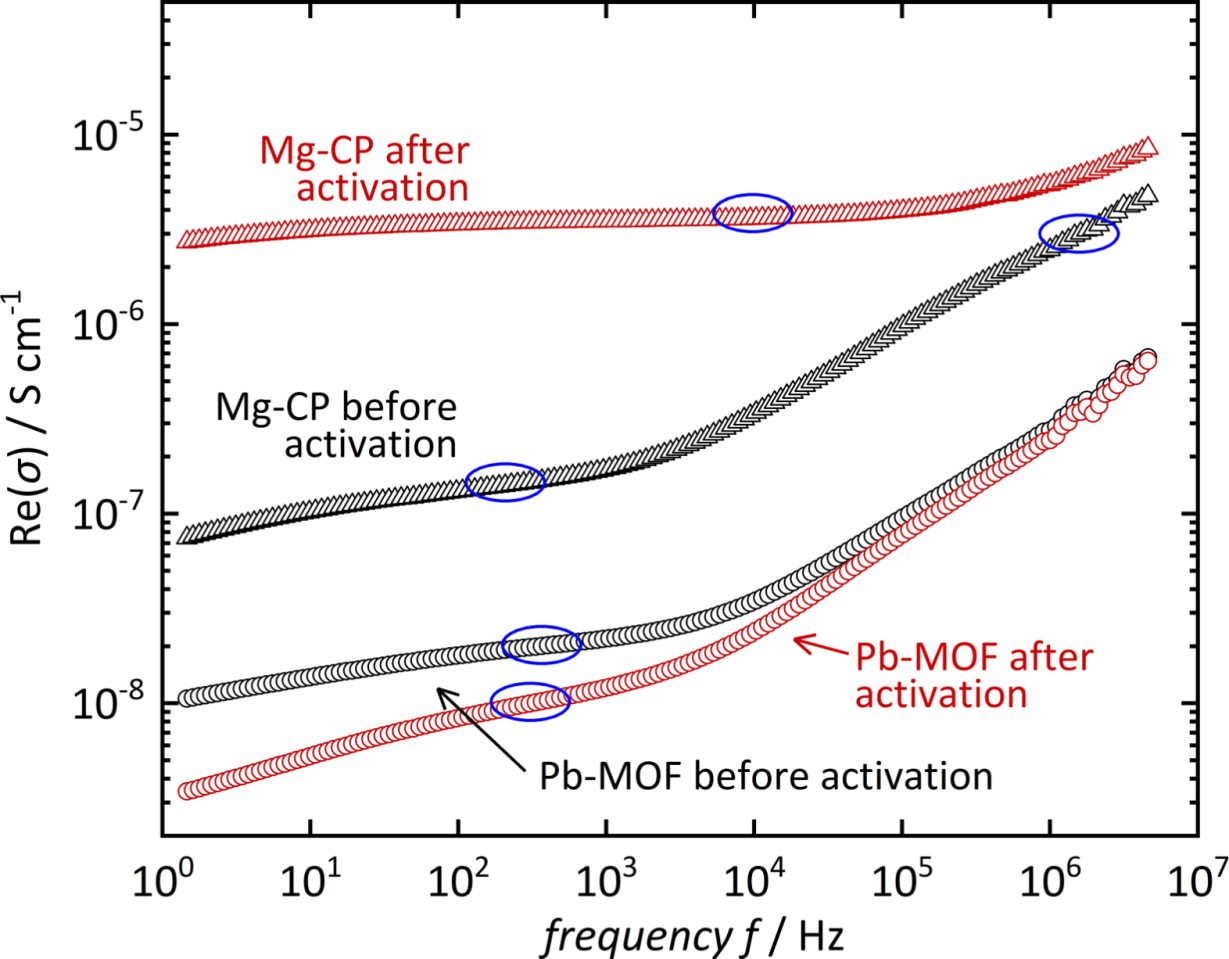
Real parts of the conductivity values of [Mg(H_2_O)_2_(H_3_L)]·H_2_O and [Pb_2_(HL)]·H_2_O obtained by conversion of impedance into permittivity (22 °C, 90% r.h.). Highlighted regions (blue) correspond to the values obtained from the Nyquist plots (see Table 1).

Another way to depict the conductivity is to plot the real part of conductivity Re(σ) vs frequency *f* [20], as shown in Figure 5. All samples show reasonably constant values in the frequency ranges that correspond to the maximum phase angles from Figure 4. These values are consistent with the conductivity values in Table 1.

**Figure 6:**
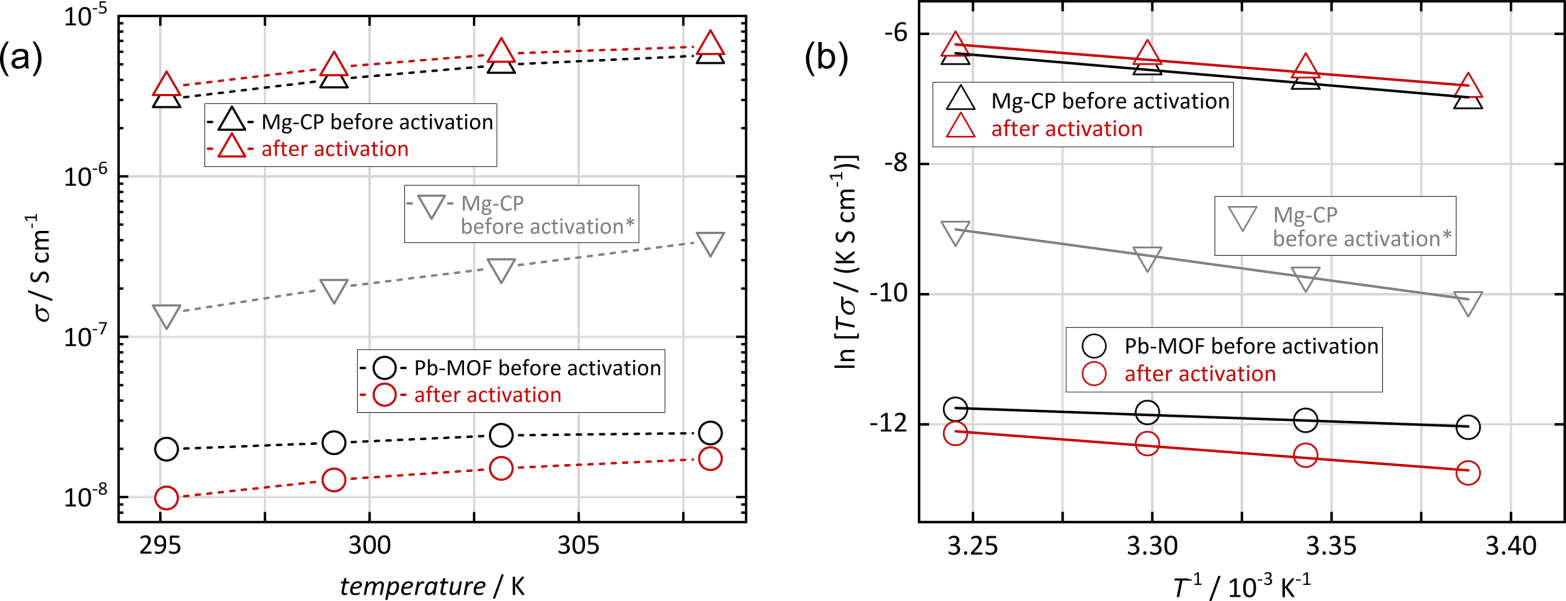
(a) Proton conductivity (dashed lines as guide to the eye) of [Mg(H_2_O)_2_(H_3_L)]·H_2_O (Mg-CP) and [Pb_2_(HL)]·H_2_O (Pb-MOF) at different temperatures (90% r.h.). (b) Arrhenius plot (with linear regression) of the same data. *The lower values for Mg-CP (gray) correspond to the conduction mode that is irreversibly erased by thermal activation.

Finally, we measured the proton conductivity at different temperatures between 22 and 35 °C (at 90% r.h.), as shown in Figure 6a. As expected, both materials show an increase in conductivity with increasing temperature; this applies to samples both before and after thermal activation. The relation between conductivity and temperature can be used to estimate the activation energy *E*_A_ of proton mobility by using the Arrhenius equation (Equation 1), where σ_0_ is a material-specific factor and *k*_B_ is Boltzmann’s constant [22–24]:


[1] $$\sigma =\frac{\sigma_{0}}{k_{\text{B}}T}\exp\left(\frac{-E_{\text{A}}}{k_{\text{B}}T}\right).$$

Figure 6b exhibits the Arrhenius plots, that is, ln(*T*·σ) vs *T*^−1^. The activation energy is obtained from the slope (−*E*_A_/*k*_B_) of the linear regression; the resulting values are shown in Table 1. For the activated samples, low values (≤0.4 eV) are observed, which indicates that proton conduction occurs predominantly by a “hopping” mechanism, that is, by formation and simultaneous cleavage of covalent bonds and hydrogen bonds between adjacent molecules, without mass transport [25]. For bulk liquid water, this is known as the Grotthuß mechanism, with reported activation energy values of 0.10–0.11 eV [26–27]. In the materials studied here, proton hopping can occur between H_2_O/H_3_O^+^ and/or deprotonated/protonated sulfonate groups (–SO_3_^−^/–SO_3_H) within the crystals. Spatial confinement may hinder proton hopping to some extent, which is why activation energies larger than in bulk liquid water are frequently observed in such materials (up to 0.4 eV) [25]. In contrast, when proton conduction occurs by mass transport (i.e., by cation diffusion of, e.g., H_3_O^+^), activation energies higher than 0.4 eV are expected [28]. This mechanism seems to dominate the additional conduction mode in the non-activated Mg-CP sample, where an activation energy of 0.64 eV is observed. This is consistent with our above assumption that this additional conduction occurs within interparticle water adsorbate layers; these liquid-like layers may facilitate molecular diffusion to some extent.

To explain the differences in proton conductivity between the (activated) Mg-CP and Pb-MOF materials, the respective crystal structures need to be considered regarding the chemical environment of the sulfonate groups and the hydrogen bonding networks of coordinating and non-coordinating water molecules. The Mg-CP network shows a clearly higher proton conductivity than Pb-MOF. As stated above, the sulfonate groups do not coordinate to Mg^2+^, but point into the interlayer regions of the layered structure, forming hydrogen bonds, both with coordinating (two per formula unit) and non-coordinating (one per formula unit) water molecules. This extensive hydrogen bonding network offers good conditions for proton hopping from one partner (H_3_O^+^, –SO_3_H) to another. In the case of the Pb-MOF material, in contrast, no such hydrogen bonding network is present. The sulfonate groups coordinate to Pb^2+^, and no hydrogen bonds other than between pairs of non-coordinating water molecules are observed, which explains that long-range proton hopping cannot occur to any significant extent in this network.

## Conclusion

The two coordination networks [Mg(H_2_O)_2_(H_3_L)]·H_2_O (Mg-CP) and [Pb_2_(HL)]·H_2_O (Pb-MOF) exhibit significantly different crystal structures, although they contain the same linker molecule, (H_2_O_3_PCH_2_)_2_-NCH_2_-C_6_H_4_-SO_3_H (H5L). Mg-CP shows a clearly higher (though altogether low) proton conductivity than Pb-MOF (by two orders of magnitude), which can be explained by said differences in crystal structure. In Mg-CP the sulfonate group does not coordinate to Mg^2+^ but is available for being part of an extended hydrogen bonding network that also includes coordinating and non-coordinating water molecules. This hydrogen bonding network seems to offer good conditions for a proton “hopping” mechanism, as confirmed by the low activation energy of the proton conductance. In Pb-MOF, on the other hand, no extended hydrogen bonding occurs, as the sulfonate groups coordinate to Pb^2+^, without forming hydrogen bonds.

## Experimental

The linker molecule (H_2_O_3_PCH_2_)_2_-NCH_2_-C_6_H_4_-SO_3_H (H_5_L, see Supporting Information File 1, Figure S1) was prepared following a published route [17]. Benzylamine was sulfonated by reaction with oleum in a first step, followed by phosphonomethylation of the amino group using phosphonic acid, formaldehyde, and hydrochloric acid. The synthesis of the compounds [Mg(H_2_O)_2_(H_3_L)]·H_2_O and [Pb_2_(HL)]·H_2_O was achieved following published procedures [17]. For [Mg(H_2_O)_2_(H_3_L)]·H_2_O, an aqueous solution of MgCl_2_ (6 mmol, 1218 mg in 2 mL H_2_O) was added to a mixture of the linker molecule (564 mg, 1.5 mmol), 1.5 mL of an aqueous solution of NaOH (*c* = 2 mol/L), 6.5 mL water, and 10 mL ethanol in a 30 mL Teflon insert, which was placed into a steel autoclave. For [Pb_2_(HL)]·H_2_O, Pb(OAc)_3_ (0.5 mmol, 189 mg in 2 mL H_2_O) was added to a mixture of the linker molecule (188 mg, 0.5 mmol), 8 mL water and 10 mL ethanol in a 30 mL Teflon insert, which was placed into a steel autoclave. Both reactors were closed and heated within 6 h to the reaction temperature of 150 °C. After 24 h, the reactors were slowly cooled down to room temperature within 12 h. The compounds were collected via filtration, dried under ambient conditions, and identified by powder X-ray diffraction. [Mg(H_2_O)_2_(H_3_L)]·H_2_O was obtained as colorless needles (240 mg, 36%) and [Pb_2_(HL)]·H_2_O as small colorless crystals (152 mg, 38%).

PXRD data at a relative humidity of 90% were recorded on a Panalytical Empyrean diffractometer equipped with a CHC plus+ chamber in a transmittance Bragg–Brentano geometry employing Cu radiation. The patterns were recorded at a temperature of 25 °C before and after the activation of sample at 80 °C and 10% relative humidity. For impedance spectroscopy, samples of the materials were pressed into cylindrical pellets using a weight of 10 t. The pellets had thicknesses of ca. 1.8 mm ([Mg(H_2_O)_2_(H_3_L)]·H_2_O) and ca. 0.5 mm ([Pb_2_(HL)]·H_2_O), respectively; their external surface area was ca. 130 mm^2^. Impedance measurements were carried out using a Novocontrol broadband dielectric spectrometer (Alpha-A High Performance Frequency Analyzer). The samples were mounted between two Novocontrol BDS1200 based blocking gold-plated electrodes, and two-wire mode measurements were made, as described in earlier studies [21,29]. The impedance data was recorded in the frequency range from 1 Hz to 4.61 MHz at an applied voltage of 0.1 V. Temperature and humidity were controlled by an Espec SH-242 climate chamber. Prior to each measurement, samples were allowed to equilibrate for 24 h.

## Supporting Information

File 1 Supplementary data.
