# Supplementary material for: The role of sulfonate groups and hydrogen bonding in the proton conductivity of two coordination networks
Source: Beilstein J Nanotechnol. 2022 May 4;13:437–43. doi: 10.3762/bjnano.13.36 (PMC9086504; doi:10.3762/bjnano.13.36)
Supplement: File 1 — Supplementary data. [file Beilstein_J_Nanotechnol-13-437-s001.pdf]

BEILSTEIN JOURNAL OF NANOTECHNOLOGY

Supporting Information

for

The role of sulfonate groups and hydrogen bonding in the proton conductivity of two coordination networks

Ali Javed, Felix Steinke, Stephan Wöhlbrandt, Hana Bunzen, Norbert Stock and Michael Tiemann

Beilstein J. Nanotechnol. **2022,** *13,* 437–443. doi:10.3762/bjnano.13.36

Supplementary data

License and Terms: This is a supporting information file under the terms of the Creative Commons Attribution License (https://creativecommons.org/licenses/by/4.0). Please note that the reuse, redistribution and reproduction in particular requires that the author(s) and source are credited and that individual graphics may be subject to special legal provisions.
The license is subject to the *Beilstein Journal of Nanotechnology* terms and conditions: (https://www.beilstein-journals.org/bjnano/terms)

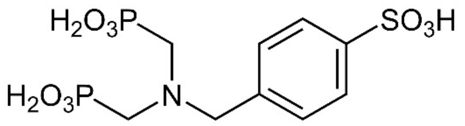

Figure S1: Linker molecule $(H_2O_3PCH_2)_2$-NCH_2-C_6H_4-SO_3H (H_5L)

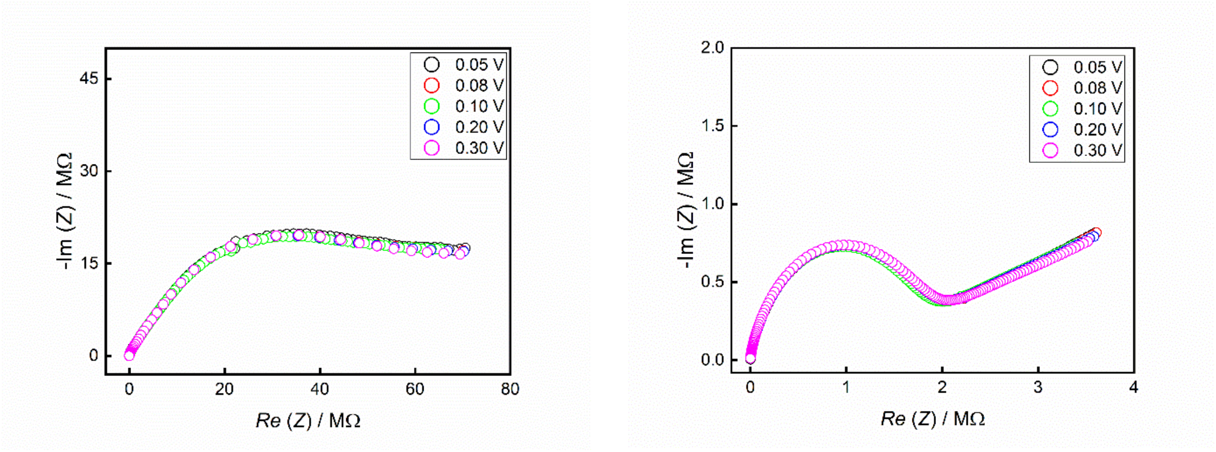

(a) (b)

Figure S2: Exemplary Nyquist plots at various applied potentials for (a) $[Mg(H_2O)_2(H_3L)]\cdot H_2O$ (22 °C, 70% r.h.) and (b) $[Pb_2(HL)]\cdot H_2O$ (22 °C, 90% r.h.).

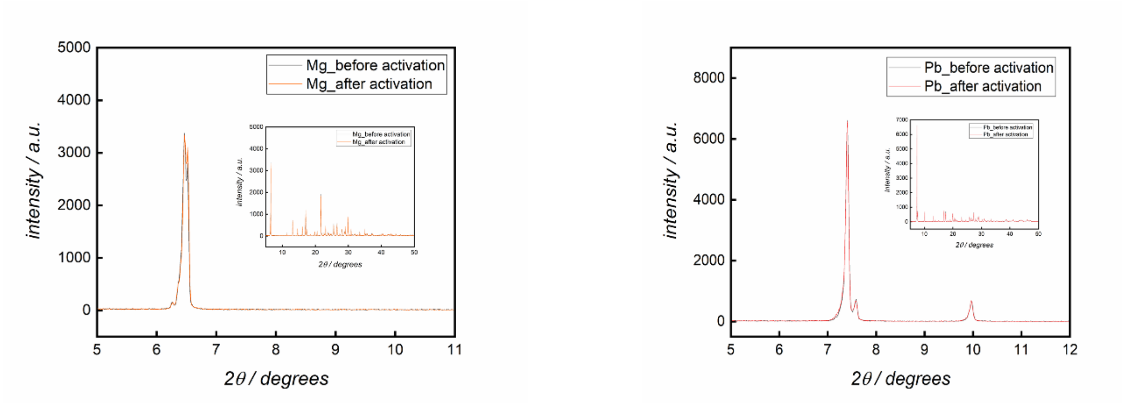

(a) (b)

Figure S3: Powder XRD patterns of (a) $[Mg(H_2O)_2(H_3L)]\cdot H_2O$ and (b) $[Pb_2(HL)]\cdot H_2O$. All measurements were performed at 25 °C and 90% relative humidity (r.h.). No significant change is observed after activation of the samples (i.e., exposure to 80 °C for 24 h).

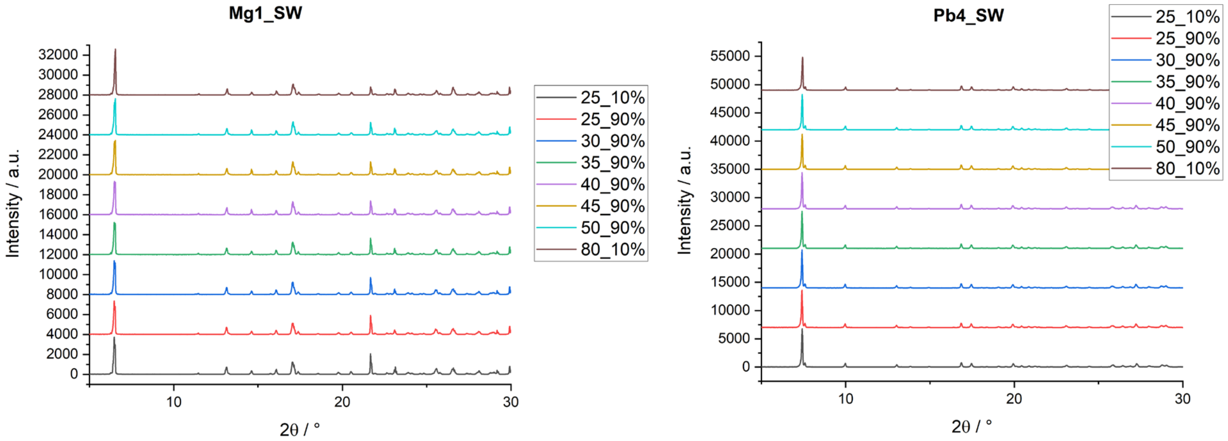

(a) **(b)**

Figure S: Powder XRD patterns of (a) $[Mg(H_2O)_2(H_3L)]\cdot H_2O$ and (b) $[Pb_2(HL)]\cdot H_2O$ at variable temperature and humidity.

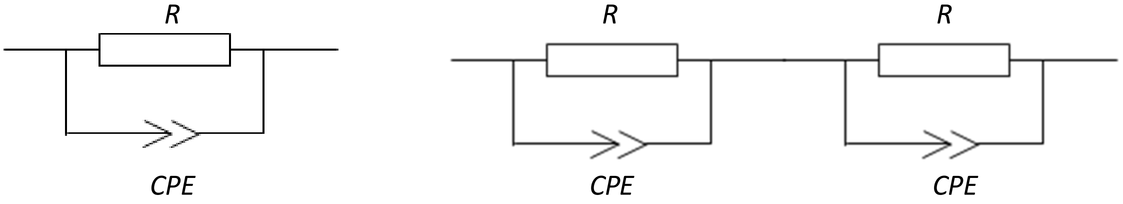

(a) **(b)**

Figure S5: Equivalent circuit models used: (a) Activated $[Mg(H_2O)_2(H_3L)]\cdot H_2O$ and for activated/non-activated $[Pb_2(HL)]\cdot H_2O$, (b) non-activated $[Mg(H_2O)_2(H_3L)]\cdot H_2O$.

Table S1: Some proton-conducting coordination networks containing sulfonate groups [1,2]

sample name	conditions	*σ* (S/cm)	E_A (eV)
$\{[Zn(bpeH)(5\text{-}sip)(H_2O)]\cdot(H_2O)\}_n$	65 °C, 95% r.h.	2.5×10^{-6}	0.54
$\{[Cu(pyz)(5\text{-}Hsip)(H_2O)_2].(H_2O)_2\}_n$	65 °C, 95% r.h.	3.5×10^{-5}	0.35
$\{[Cu(bpee)_{0.5}(5\text{-}sip)(H_2O)_2]\cdot(H_2O)_4(bpeeH_2)_{0.5}\}_n$	65 °C, 95% r.h.	9.9×10^{-8}	0.40
$\{[Cu(bpy)(5\text{-}Hsip)(H_2O)]\cdot(H_2O)_2\}_n$	65 °C, 95% r.h.	5.8×10^{-6}	0.43
$\{[Cu(bpy)_2(5\text{-}H_2sip)_2]\cdot(H_2O)_6\}_n$	65 °C, 95% r.h.	1.4×10^{-6}	0.45
$\{[Cu_2(sba)_2(bpg)_2(H_2O)_3]\_5H_2O\}_n$	80 °C. 95% r.h.	9.4×10^{-3}	0.64
$Cu_4(5\text{-}sip)_2(OH)_2(DMF)_2$	95 °C. 95% r.h.	7.4×10^{-4}	1.32
$UiO\text{-}66(SO_3H)_2$	80 °C. 90% r.h.	8.4×10^{-2}	0.32
$MIL\text{-}101\text{-}SO_3H$	70 °C. 90% r.h.	4.3×10^{-5}	0.27

References

1. Liu, R.; Wang, D.-Y.; Shi, J.-R.; Li, G. *Coord. Chem. Rev.* **2021,** *431,* 213747. doi:10.1016/j.ccr.2020.213747
2. Lim, D.-W.; Kitagawa, H. *Chem. Rev.* **2020,** *120,* 8416. doi:10.1021/acs.chemrev.9b00842
